# Reporting of test comparisons in diagnostic accuracy studies: A literature review

**DOI:** 10.1002/jrsm.1469

**Published:** 2020-12-10

**Authors:** Yasaman Vali, Bada Yang, Maria Olsen, Mariska M. G. Leeflang, Patrick M. M. Bossuyt

**Affiliations:** ^1^ Department of Epidemiology and Data Science, Amsterdam UMC University of Amsterdam Amsterdam The Netherlands

**Keywords:** comparative accuracy studies, diagnostic accuracy, reporting, test comparison

## Abstract

Comparative accuracy studies evaluate the relative performance of two or more diagnostic tests. As any other form of research, such studies should be reported in an informative manner, to allow replication and to be useful for decision‐making. In this study we aimed to assess whether and how components of test comparisons were reported in comparative accuracy studies. We evaluated 100 comparative accuracy studies, published in 2015, 2016 or 2017, randomly sampled from 238 comparative accuracy systematic reviews. We extracted information on 20 reporting items, pertaining to the identification of the test comparison, its validity, and the actual results of the comparison. About a third of the studies (*n* = 36) did not report the comparison as a study objective or hypothesis. Although most studies (*n* = 86) reported how participants had been allocated to index tests, we could often not evaluate whether test interpreters had been blinded to the results of other index tests (*n* = 40; among 59 applicable studies), nor could we identify the sequence of index tests (*n* = 52; among 90 applicable studies) or the methods for comparing measures of accuracy (*n* = 59). Two‐by‐four table data (revealing the agreement between index tests) were only reported by 9 of 90 paired comparative studies. More than half of the studies (*n* = 64) did not provide measures of statistical imprecision for comparative accuracy. Our findings suggest that components of test comparisons are frequently missing or incompletely described in comparative accuracy studies included in systematic reviews. Explicit guidance for reporting comparative accuracy studies may facilitate the production of full and informative study reports.

*What is already known?*

- Transparent reporting of studies is essential to allow readers to appreciate study findings and limitations.

*What is new?*

- Comparative accuracy studies (a specific type of diagnostic accuracy studies that evaluate and compare the accuracy of two or more tests) frequently fail to report, or incompletely report information about comparisons of index tests that helps study identification, validity assessment, and interpretation of results.

*Potential impact for RSM readers?*

- Incomplete reporting of comparative accuracy studies will complicate their appraisal and synthesis in diagnostic test accuracy systematic reviews. There is a clear need for more informative study reports, which could be facilitated through the development of explicit reporting guidelines specifically for comparative accuracy studies.

## 1 INTRODUCTION

Diagnostic accuracy studies provide information on the performance of a test in accurately distinguishing individuals with and without a target condition. Such studies can be focused on a single index test, but they can also evaluate two or more index tests for detecting the same target condition and compare their accuracy.^1^, ^2^ Well‐designed comparative accuracy studies can provide valuable evidence to clinicians and policy‐makers, helping them to select the optimal test for patients among competing tests.

As other clinical studies, comparative accuracy studies should be reported in an informative and reproducible way to allow the reader to evaluate the validity of the study, to appreciate the study findings, and to consider their applicability to other patient groups and settings.^3^, ^4^, ^5^, ^6^ Deficiencies in reporting not only can lead to incorrect conclusions and make decision‐making difficult, it is also a source of avoidable research waste and, as such, a threat to evidence‐based medicine.^7^, ^8^

While all diagnostic accuracy studies need to be transparently reported, comparative accuracy studies face an added reporting challenge. Authors of comparative accuracy studies should not only report details of each index test under investigation, but also describe how the index tests were compared to each other. They have to specify the design and methodology of their comparison in a transparent and reproducible manner and report the comparative accuracy results in such a way that statistical inference regarding the relative performance of the tests is possible.

Existing guidance for reporting diagnostic studies has no specific instructions for comparative accuracy studies.^3^ Previous evaluations of the informativeness of reports of diagnostic accuracy studies largely focused on single test evaluations, without targeting comparative accuracy studies.^9^, ^10^ We evaluated published reports of recent comparative accuracy studies to evaluate whether and how components of test comparisons were described.

## 2 OBJECTIVES

We aimed to examine the reporting characteristics of comparative accuracy studies and assess whether information on identifying the comparison, aspects of validity, and results of the comparison were adequately reported.

## 3 METHODS

### 3.1 Study design

This study is a literature survey of comparative accuracy studies. The study protocol was made available through the Open Science Framework (https://osf.io/72xpy).

### 3.2 Data sources

For the purpose of this study, comparative accuracy studies were sampled from studies included in systematic reviews that had compared the accuracy of two or more tests. We selected these systematic reviews from an existing overview of 238 comparative accuracy systematic reviews published between 2017 and 2018.^11^ Briefly, the overview included all systematic reviews including a comparison between the accuracy of index tests indexed in MEDLINE in 2017. The search strategy for this overview is provided in Table S1 in Data S1.

### 3.3 Eligibility criteria and study selection

Eligible were all comparative accuracy studies on humans. We defined a comparative accuracy study as a study that (1) evaluates the accuracy of two or more index tests and (2) for which the published study report contains at least one statement in which the accuracy of these index tests is being compared. For assessing whether such a statement is present, we looked for comparative language, that is, terms as “comparison”, “comparative”, “higher/lower”, “superior/inferior”, “better/best/worst/worse”, and “more/most”.

One reviewer retrieved all references to primary studies included in the 238 systematic reviews. We then applied a filter based on year of publication. We restricted inclusion to comparative accuracy studies published in 2015, 2016, or 2017, to evaluate recent practice. From the primary studies of these 3 years, we randomly selected published study reports and evaluated eligibility. Each study was assigned a random study number, using a random number generator on Google Sheets software (Google, Mountain View, California, U.S.). The studies were evaluated for eligibility starting with the lowest study numbers, until 100 primary comparative accuracy studies were included. As there is no widely accepted sample size calculation for our type of methodological review, the authors agreed to sample 100 studies for feasibility reasons. Evaluation of eligibility was done in two steps: first based on the title and abstract, and then the full‐text article. Each study report was assessed by two independent assessors for eligibility. Disagreements were resolved by consensus, or by consulting a senior author. We excluded non‐English language studies.

### 3.4 Data extraction

For each comparative accuracy study, two independent assessors looked for key reporting items regarding comparisons in the body of the full‐text report. Study abstracts were not assessed. In the absence of a specific reporting guidance for comparative accuracy studies, we developed a set of items which we deemed specifically relevant to comparative accuracy studies (Table S2 in Data S1). An initial list of potentially relevant items was developed by two authors (Y.V. and B.Y.) through brainstorming and consultation of existing reporting guidelines. This list was subsequently reviewed and revised iteratively by the co‐authors. The items in the final list were largely adapted from the items in STARD 2015 (reporting guidelines for diagnostic accuracy studies),^3^ many of which were directly applicable to comparative accuracy studies (See Table S2 in Data S1 for the source of each item). In addition, the QUADAS‐C Delphi study for developing a risk of bias tool for comparative accuracy studies produced a number of items related to potential bias.^12^ Lastly, items from CONSORT 2010 (reporting guideline for parallel group randomized trials) were adapted for comparative accuracy studies that use random allocation.^13^

A comparative accuracy study may contain more than one test comparison. Some studies evaluate large numbers of index tests, with the possibility of presenting numerous comparisons. We therefore made an additional restriction, by focusing exclusively on the first comparison reported in the article. For example, if a study had evaluated five index tests and reported 10 pairwise comparisons, one by one, the first pair reported would be considered to be the first comparison.

### 3.5 Data analysis

We used descriptive statistics to summarize the results. We presented the number of studies that reported a particular item, accompanied by examples how these items were reported (if applicable).

## 4 RESULTS

### 4.1 Search results

**FIGURE 1:**
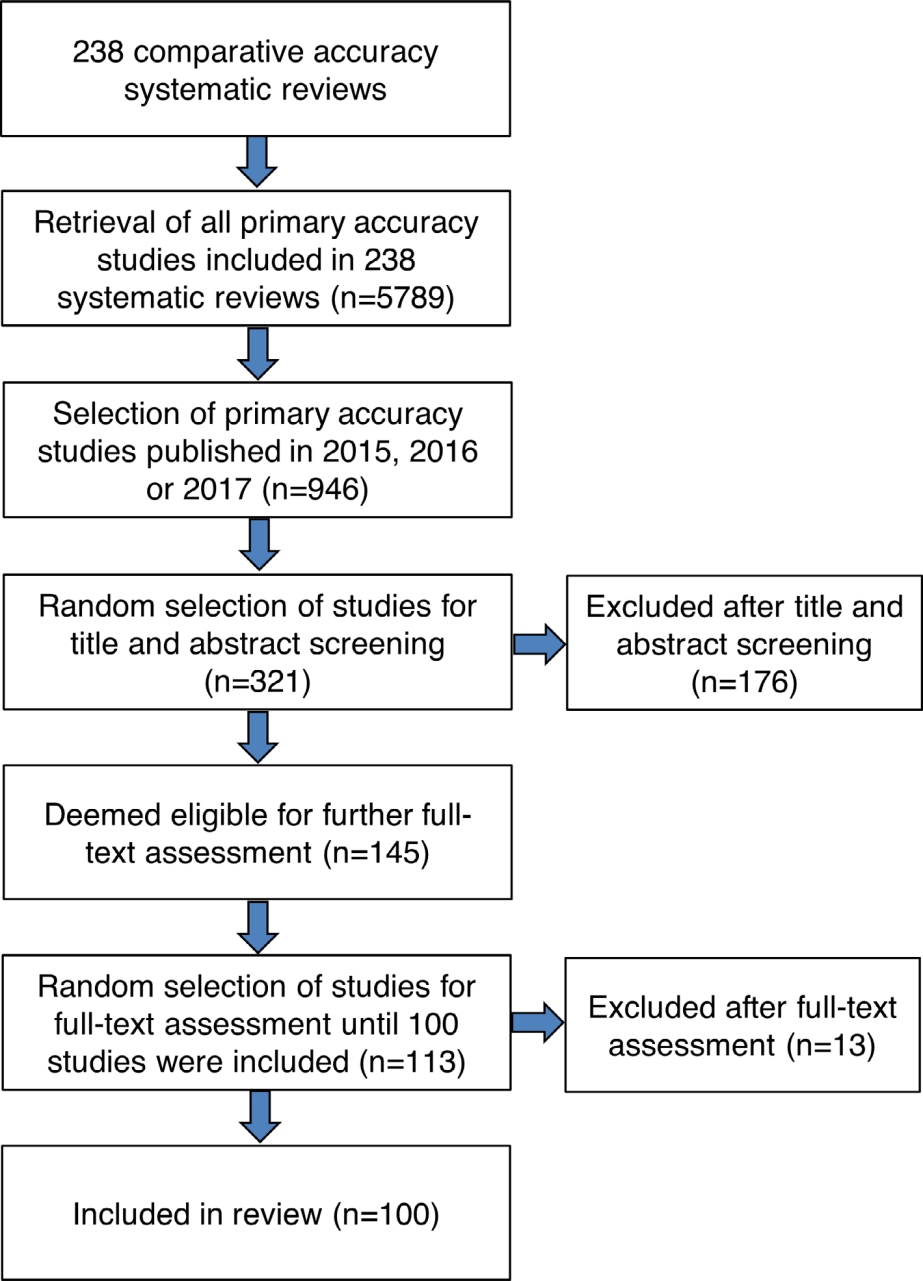
Flow diagram of included studies [Colour figure can be viewed at wileyonlinelibrary.com]

From the 238 systematic reviews of comparative accuracy, we retrieved 5789 references to primary accuracy studies, of which 946 were published in 2015, 2016, or 2017. We assigned a random number to each of the 946 primary studies and selected an arbitrary number of 321 studies with the lowest assigned numbers for title and abstract screening. We then excluded 176 of 321 studies during this phase. For the remaining 145 studies, we assessed full text articles in random order until we included 100 comparative accuracy studies. Eventually, we assessed the first 113 full text articles in order to include 100 studies (Figure 1).

### 4.2 Characteristics of included studies

The characteristics of included comparative accuracy studies are described in Table 1. Fifty‐nine studies were published in 2015, 36 studies in 2016, and 5 studies in 2017. A wide range of target conditions were evaluated, the most frequent being neoplasms (*n* = 54), digestive system disorders (*n* = 14), and infectious diseases (*n* = 10). In almost half of the studies, the index tests in the comparisons were biochemical tests (*n* = 50) and imaging modalities (*n* = 47). In 49 studies the first comparison reported in the article was between two index tests, while in the other 47 studies the first comparisons consisted of three or more index tests. This was unclear in four studies.

**TABLE 1:** Characteristics of 100 included comparative accuracy studies

Characteristic	Total (N = 100)
**Publication year**	
2015	59
2016	36
2017	5
**Target condition**	
Neoplasms	54
Digestive system	14
Infectious	10
Musculoskeletal/connective tissue	8
Mental/behavioral/neurodevelopmental	4
Circulatory system	4
Other	6
**Type of index test** ^**a**^	
Biochemical	50
Imaging	47
Pathology	6
Combination of multiple types	4
Questionnaire	3
Clinical	3
**Number of index tests in the first comparison**	
2	49
3	17
4	15
5 or more	15
Unclear	4

^a^ There can be multiple types of index tests per study.

### 4.3 Reporting of components of a test comparison

#### 4.3.1 Identifying the comparison

We summarized reporting items that could help to identify comparative accuracy studies in Table 2. The comparative nature of the study could not be identified in the titles of most of the studies (*n* = 73). Of those that identified their study as a comparative accuracy study in the title (*n* = 27), 6 used a study label indicating a comparison (such as “comparative study” or “prospective randomized study”) and 21 studies indicated the comparison otherwise (e.g., using comparative language such as “superior” or “outperformed”). More than half of the studies reported the test comparison as part of their objectives (*n* = 60) or hypothesis (*n* = 3) or both (*n* = 1), while about a third (*n* = 36) did not mention test comparison as a specific objective or hypothesis.

**TABLE 2:** Frequency of studies that reported a particular item for identifying the comparison

Reporting items	Total (N = 100)
Identification of the study as a comparative accuracy study in the title	
Yes, by implying that there was a comparison	21
Yes, by using a study design label	6
No	73
Reporting test comparison as an objective (or stating a hypothesis regarding a comparison)	
Yes, as objective	60
Yes, as hypothesis	3
Both	1
No	36
Reporting which index tests are exactly being compared, before the paper's results section	
Yes	66
No	34

The majority (*n* = 66) stated which index tests were being compared, before reporting the performance of the tests in the results section of the article. In the remaining 34 studies, the index tests within the comparison were found after the methods section: in the results, tables, figures, or discussion section.

#### 4.3.2 Information relevant to the validity of the comparison

Table 3 summarizes the results for items related to the validity of the comparison. In 16 studies including composite index tests, only 5 reported the criterion for test positivity. The majority of studies did not report whether participants were either consecutively or randomly sampled (*n* = 62). Only 34 studies reported this explicitly by using the words “consecutive” or “random”; four studies reported this by describing the sampling process.

**TABLE 3:** Frequency of studies that reported a particular item relevant to the validity of the comparison

Reporting items	Total (N = 100)
**Participant sampling and allocation**	
Reporting whether participants were either consecutively or randomly sampled	
Yes, explicitly reported	34
Yes, inferable from description	4
No	62
Reporting how participants were allocated to different index tests	
Yes, explicitly reported	34
Yes, inferable from description	52
No	14
If randomization was used, reporting the method used to generate the random allocation sequence	
Yes	1
No	0
Not applicable	99
If randomization was used, reporting whether allocation was concealed	
Yes	0
No	1
Not applicable	99
**Test methods**	
If composite index tests were used, reporting the criterion for test positivity	
Yes	5
No	11
Not applicable; no composite index tests	84
If participants received multiple index tests, reporting whether the index test interpreters were blinded to the other index test results	
Yes, the word “blinding” or a description of blinding is provided	7
Yes, inferable from description	12
No	40
Not applicable (unclear whether paired (*n* = 6), clearly not a paired design (*n* = 4), or objective tests (*n* = 31))	41
If participants received multiple index tests, reporting the sequence of index tests performed on each participant	
Yes, reports that there was a fixed test order, or that the tests were performed simultaneously	37
Yes, reports that there was a different test order for two groups	1
No	52
Not applicable (unclear whether paired (*n* = 6), or clearly not a paired design (*n* = 4))	10
Reporting the time interval between the index tests	
Yes, explicitly reported	15
Yes, inferable from description	38
No	47
If two or more reference standards were used, reporting how reference standards were chosen for a participant	
Yes, choice was dependent on index test results	2
Yes, choice was dependent on a third test not in the comparison	3
Yes, choice was based on clinical indication	1
No	12
Not applicable (single reference standard (*n* = 72), or unclear how many reference standards were used (*n* = 10))	82
**Analysis**	
Reporting methods for comparing diagnostic accuracy	
Yes	41
No	59
**Participant flow and characteristics**	
Reporting a participant flow diagram	
Yes, includes all index tests	9
Yes, but includes none of the index tests	7
No	84
If a study was not fully paired, reporting the baseline characteristics (at least age and gender) of participants	
Yes, for each index test	2
Yes, but only for the entire study group	2
No	2
Not applicable, (fully paired (*n* = 79) or unclear whether fully paired (*n* = 15))	94

In 14 studies, it was not clear how the participants were allocated to each index test. Of the 86 studies that described the allocation method only 34 did so explicitly (e.g., by stating “participants were screened using all three cognitive measures”^14^), while for 52 studies this was inferable from a description provided in the methods, or from figures or tables.

We found 90 studies with a paired design, in which a participant received two or more index tests (for nine of these studies the allocation was not clearly reported, but it became clear that at least some participants received multiple index tests based on other descriptions in the methods or results section). In such paired studies, each index test should ideally be interpreted blinded from the other index test results, if the index test involves subjective interpretation. We judged that one or more index tests in the comparison may involve subjective interpretation in 59 paired studies. However, only 19 of 59 studies reported whether blinding was implemented, by using the word “blinding” or similar wording (*n* = 7), for example by declaring that interpreters were not aware of other test results,^15^ or by describing the study process (*n* = 12). An example of the latter was a comparison of endoscopy techniques, each interpreted by a single endoscopist.^16^ Information on blinding was missing from 40 of 59 study reports.

Only 38 of the 90 paired studies reported the sequence of index tests performed on each participant. Most often there was a fixed order for all participants. For example, by reporting the exact sequence of three cognitive tests performed on each participant.^14^ One study reported a fixed test order for one subgroup and a reversed order for a second subgroup.^17^

The time interval between the index tests was not specified in approximately half of the study reports (*n* = 47). Of 53 studies that reported this item, 15 described it explicitly, for example, by reporting the median number of days. For 38 studies this was inferable from other information in the study report. An example of the latter was a study in which all biomarkers were tested in the same blood sample.^18^

The majority of studies failed to report the time interval between the index tests and the reference standard (*n* = 69). One study reported this only for one of the two index tests, while the other 30 studies stated this for each of the index tests in the comparison.

Ideally, a single, preferred (best available) reference standard should be used to verify all index test results. In 18 studies, two or more reference standards were used. We examined in these 18 studies how the reference standard was chosen for each participant. Six studies reported that the choice depended on index test results (*n* = 2), on a third test not in the comparison (*n* = 3), or on clinical indication (*n* = 1). In 12 studies, the choice for the reference standard was not explained.

We examined whether methods for comparing diagnostic accuracy estimates, statistical or other, were described in the article (e.g., McNemar's test statistic for paired data, or tests for differences in the area under the receiver operating characteristic [ROC] curve). Such methods were reported only by a minority of studies (*n* = 41), and absent in the 59 other study reports.

Of all 100 evaluated studies, only 16 provided a participant flow diagram. Of these, seven studies did not include any of the index tests in the flow diagram.

When studies were not fully paired, that is, partially paired or unpaired (*n* = 6), we examined whether studies reported baseline characteristics of participants (at least age and gender). Two studies reported baseline characteristics for each index test group, thereby allowing the reader to assess the comparability of the index test groups. However, the remaining studies either reported baseline characteristics for the entire group of participants (*n* = 2) or did not report them at all (*n* = 2).

#### 4.3.3 Results of the comparison

Table 4 shows the number of comparative accuracy studies that reported information on the results of the actual comparison. Not all studies reported sufficient data for construction of two‐by‐two tables (index test against reference standard). Such data were reported by only 35 studies; 33 studies provided data for each index test in the comparison, while two studies provided data for only some of the index tests.

**TABLE 4:** Frequency of studies that reported a particular item relevant to the results of the comparison and limitations

Reporting items	Total (N = 100)
**Contingency table data**	
Reporting the two‐by‐two contingency table data	
Yes, for each index test	33
Yes, but only for some of the index tests	2
No	65
If participants received multiple index tests, reporting the two‐by‐four contingency table data	
Yes	9
No	81
Not applicable (unclear whether paired (*n* = 6), or clearly not a paired design (*n* = 4))	10
**Comparative accuracy estimates**	
Reporting the results using comparative accuracy measures	
Yes, difference in sensitivity and difference in specificity	2
Yes, difference in area under the curve	1
Yes, odds ratio of sensitivity and odds ratio of specificity	1
No	96
Reporting measures of precision for comparative accuracy	
Yes, *p*‐values	33
Yes, confidence intervals	1
Yes, both	2
No	64
**Limitations**	
Reporting any limitations regarding the comparison	
Yes	18
No	82

We also examined whether data for the construction of two‐by‐four tables^19^ (tables that cross‐classify the results of two index tests being compared within diseased and non‐diseased participants; Table 5) were reported by paired accuracy studies. This was the case in only 9 of 90 paired studies.

**TABLE 5:** Joint classification of index tests and reference standard results in a paired design (also called two‐by‐four table), adapted from Reference 19

	Reference standard positive			Reference standard negative		
	Test B +	Test B −	Total	Test B +	Test B −	Total
Test A +	*a*	*b*	*a+b*	*e*	*f*	*e+f*
Test A −	*c*	*d*	*c+d*	*g*	*h*	*g+h*
Total	*a+c*	*b+d*	*n*_*+*_	*e+g*	*f+h*	*n*_*−*_

Only four studies reported their findings using a measure of comparative accuracy. Reported measures were difference in sensitivity and difference in specificity (*n* = 2), odds ratio of sensitivity and odds ratio of specificity (*n* = 1) or difference in the area under the ROC curves (*n* = 1). The others (*n* = 96) did not report measures of comparative accuracy. Expressions of statistical uncertainty for the comparisons were reported in 36 studies, either as p‐values (*n* = 33), confidence intervals (*n* = 1) or both (*n* = 2). The 64 other studies reported the comparison without statistical uncertainty, while 10 of those studies explicitly reported that they used a statistical method for comparing diagnostic accuracy estimates.

Potential limitations regarding the test comparison were mentioned in a minority of study reports (*n* = 18). For example, one study that compared the accuracy of 22‐gauge versus 25‐gauge needles for diagnosing pancreatic tumors reported that the endoscopists were not blinded to needle type.^20^ In another study that compared the accuracy of two MRI‐based scoring systems for prostate cancer diagnosis, authors admitted that MRI readers assigned both scores in a single session, thereby introducing the possibility that scores for one scheme could have influenced the other.^21^ Although we expected from even an almost perfect study to discuss at least one potential or true limitation, 82 studies did not mention any such limitations.

## 5 DISCUSSION

### 5.1 Summary of findings

Well‐conducted comparative accuracy studies, while scarce, have the potential to yield high‐certainty evidence for informing clinical decision making regarding tests.^1^ Considering their importance, they should be reported in sufficient detail, to allow readers to appreciate their findings.

Our findings suggest that the reporting of items to identify the comparison, to assess the validity, and to interpret the results of the comparison is suboptimal in comparative accuracy studies. Many of the items were missing or incompletely reported. Even when information on a particular item could be identified, such as allocation method, this could often only be inferred indirectly, from more general descriptions of study processes, or tables and figures.

Measures of comparative accuracy were rarely used, and expressions of statistical uncertainty were not available in the majority of study reports. Thus, it was often not clear how the studies had come to the given conclusion about which test was more/most accurate and we suspect that the conclusions were often simply based on the separate accuracy estimates for the respective tests.

### 5.2 Strengths and limitations

Previous evaluations have highlighted incomplete and ambiguous reporting of diagnostic accuracy studies,^9^ but few studies have focused on comparisons of tests. A recently published methodological survey showed poor reporting of systematic reviews that had evaluated and compared multiple tests.^22^ To our knowledge, no previous study assessed the reporting of information on test comparisons in comparative accuracy studies.

Our review has a number of limitations. First, there is currently no agreed definition of comparative accuracy studies. The definition used in this survey is a “multiple test accuracy study with a comparative statement”, which others may disagree with. Second, given our limited sample size of 100 studies and sampling method (we only included comparative accuracy studies included in systematic reviews), results from an independent sample of comparative accuracy studies may differ. However, since systematic review authors may have selected studies for inclusion when reporting was sufficient, the number of studies in our sample that reported a particular item (e.g., identification as a comparative study in the title) may even be overestimated rather than underestimated. Third, there may be inherent subjectivity in the assessment of specific reporting items. For instance, a seemingly straightforward question as “was the allocation method reported” was difficult to answer when the authors did not explicitly describe the allocation process. This required a judgment as to whether the different pieces of information in the study report were sufficient to ascertain the allocation method. To minimize subjectivity, we used assessment in duplicate and had internal discussions. Lastly, we assessed the reporting in relation to only the first comparison of each study. Although unlikely, we cannot exclude the possibility that subsequent comparisons were more completely reported.

### 5.3 Interpretation of findings

The potential implications of poor reporting are wide and serious. Those that search for comparative accuracy studies may spend excessive time and effort due to the lack of appropriate identifiers. The absence of an explicit description of the study design may impede attempts at evaluating the risk of bias of such studies. Incomplete reporting of the results of test comparisons may complicate the incorporation of study data in evidence syntheses and may lead to misinterpretation when translating study findings to clinical practice.

Although we can merely speculate on the causes of poor reporting, one possibility is a low overall awareness of the importance of test comparisons in diagnostic accuracy studies. Investigators may believe that diagnostic accuracy studies (even when multiple index tests are included) should mostly aim to provide reliable estimates of the accuracy of individual tests. Many authors did not state the comparison as a study objective or hypothesis; this was the case in 36 studies in our sample. Despite this, authors still compared the accuracy of index tests and made a comparative statement. We believe that even these casual comparisons should be based on solid evidence. Making comparative statements without a proper description of the study features on which the comparison is based can increase the risk of “spin” or over‐interpretation of the results. Over‐interpretation and misreporting of results are frequent in diagnostic accuracy studies^23^, ^24^ and using exaggerated language in comparative accuracy studies may not only result in research waste but also cause harm to patients by misleading clinicians to select inappropriate tests.

Another possibility is that investigators are less aware of potential sources of bias in test accuracy comparisons, and therefore could not know which items were essential to report. This would be unsurprising, since methodological research on bias in test accuracy comparisons is limited, compared to numerous publications and instruments to identify sources of bias in estimates of accuracy of a single test.^25^, ^26^, ^27^

Authors should aim to improve the quality of reporting in their studies, by becoming more familiar with different aspects of comparative study designs and appropriately interpreting their findings. Yet they are not the only ones bearing responsibility. To ensure accurate interpretation and dissemination of research, editors and peer reviewers should also be held accountable for identifying any comparative statements and for critically examining whether such claims can be supported by the study design and results.

Providing authors with guidance may help them to improve the reporting of their studies. The STARD statement, which was developed in 2003 and updated in 2015,^3^, ^28^ is the established reporting guideline for all types of diagnostic accuracy studies. While STARD includes reporting items pertaining to test comparisons, it also lacks some key items, such as participant allocation method. STARD could be extended to address these additional comparative items, and a revised explanation document could highlight the importance of explicit and rigorous test comparisons.

## 6 CONCLUSIONS

Information about comparisons of index tests that helps study identification, validity assessment, and interpretation of results is missing or incompletely reported in comparative accuracy studies included in systematic reviews. This illustrates a clear need for improvement in the standards of reporting for comparative accuracy studies. Better reporting could be facilitated through the development of explicit reporting guidelines specifically for comparative accuracy studies.

## CONFLICT OF INTEREST

All authors declare that there are no conflicts of interest.

## AUTHOR CONTRIBUTIONS

Y.V.: Conceptualization, Project administration, Methodology, Investigation, Formal analysis, Writing – Original Draft, Writing – Review & Editing. B.Y.: Conceptualization, Methodology, Investigation, Formal analysis, Writing – Original Draft, Writing – Review & Editing. M.O.: Methodology, Investigation, Writing – Review & Editing. M.M.G.L.: Methodology, Writing – Review & Editing, Supervision. P.M.M.B.: Methodology, Writing – Review & Editing, Supervision.

## Supporting information


**Data S1.** Supporting information.
**Table S1.** Search strategy for the overview of comparative accuracy systematic reviews.
**Table S2.** Reporting items for comparative accuracy studies developed for this literature review.
**Table S3.** List of included comparative accuracy studies.

## Data Availability

The data that support the findings of this study are available from the corresponding author upon reasonable request.
