# Supplementary material for: Reporting of test comparisons in diagnostic accuracy studies: A literature review
Source: Res Synth Methods. 2020 Dec 10;12(3):357–67. doi: 10.1002/jrsm.1469 (PMC8246725; doi:10.1002/jrsm.1469)
Supplement: Supplementary file 1 — Data S1. Supporting information. Table S1. Search strategy for the overview of comparative accuracy systematic reviews. Table S2. Reporting items for comparative accuracy studies developed for this literature review. Table S3. List of included comparative accuracy studies. [file JRSM-12-357-s001.docx]

# Supplementary documents

**Table S1. Search strategy for the overview of comparative accuracy systematic reviews**

MEDLINE (Ovid interface) search strategy on February 15, 2018

| #1 | (MEDLINE or systematic review or (literature adj2 review)).tw. or meta-analysis.pt. or meta-analysis as topic/ or meta-analys*.ti,ab,kf. or (search* adj12 (literature or database?)).ti,ab |
| --- | --- |
| #2 | exp "sensitivity and specificity"/ or exp "mass screening"/ or "reference values"/ or "false positive reactions"/ or "false negative reactions"/ or specificit$.tw. or screening.tw. or false positive$.tw. or false negative$.tw. or accuracy.tw. or predictive value$.tw. or reference value$.tw. or roc$.tw. or likelihood ratio$.tw. |
| #3 | 1 and 2 |
| #4* | limit 3 to yr="2015 -Current" |
| #5 | (case report* or letter* or historical article or "review of reported case*" or editorial* or comment*).ti,ab,kf. |
| #6 | limit 4 to (case reports or comment or editorial or letter) |
| #7 | 4 not (5 or 6) |

*For our overview, only records published in 2017 were screened for eligibility**.**

**Table S2. Reporting items for comparative accuracy studies developed for this literature review.**

| **Reporting item** | **Explanation** | **Source** |
| --- | --- | --- |
| **Identifying the comparison** | | |
| 1. Identification of the study as a comparative accuracy study in the title | Comparative studies should be easily discernable from single test accuracy studies. | Adapted from item 1 of STARD 2015 (1) |
| 1. Reporting test comparison as an objective (or stating a hypothesis regarding a comparison) | Informs the reader that the purpose of the study is to compare test accuracy. | Adapted from item 4 of STARD 2015 (1) |
| 1. Reporting which index tests are exactly being compared, before the paper’s results section | Studies may assess a multitude of index tests and include multiple comparisons. Making clear which comparisons are of interest may discourage selective reporting of results. | Item generated for this review |
| **Information relevant to the validity of the comparison** | | |
| ***Participant sampling and allocation*** | |  |
| 1. Reporting whether participants were either consecutively or randomly sampled | A comparison can only be valid if done in the right participants. A sample that is not representative of the population is likely to bias the comparison. | Adapted from item 9 of STARD 2015 (1) |
| 1. Reporting how participants were allocated to different index tests | In order to assess whether the index test groups are comparable regarding factors that may affect accuracy, studies should report the allocation method. This could be pairing (the index tests are performed in the same participant), randomization, or otherwise. | Item generated for this review |
| 1. If randomization was used, reporting the method used to generate the random allocation sequence | For successful randomization, a truly random and unpredictable allocation sequence should be used. | Adapted from item 8a of CONSORT 2010 (2) |
| 1. If randomization was used, reporting whether allocation was concealed | For successful randomization, the random sequence should be concealed until participants are allocated. | Adapted from item 9 of CONSORT 2010 (2) |
| ***Test methods*** | |  |
| 1. If composite index tests were used, reporting the criterion for test positivity | Comparisons in which test A is compared with a composite test of A and B may occur. A clear definition of how A and B are combined to produce a test result may not always be reported. | Item generated for this review |
| 1. If participants received multiple index tests, reporting whether the index test interpreters were blinded to the other index test results | The comparison is at risk of bias if index test results are interpreted with knowledge of other index test results. | Item based on results from the QUADAS-C Delphi study (3) |
| 1. If participants received multiple index tests, reporting the sequence of index tests performed on each participant | Readers should be able to assess whether one index test could have influenced the performance of subsequent index test(s). | Item based on results from the QUADAS-C Delphi study (3) |
| 1. Reporting the time interval between the index tests | The comparison may not be valid if the index tests are not performed at the same disease stage. For diagnostic tests that rely on samples, we defined the ‘timing of test performance’ to equal the timing of sample collection, , unless it was clearly reported that competing tests were measured using the same sample but in different time points. | Item based on results from the QUADAS-C Delphi study (3) |
| 1. If two or more reference standards were used, reporting how reference standards were chosen for a participant | Ideally, the disease status of all participants should be verified by a single, preferred reference standard. If two or more reference standards are used, the risk of bias depends on how a reference standard was chosen for each participant. | Item generated for this review |
| ***Analysis*** | |  |
| 1. Reporting methods for comparing diagnostic accuracy | The study should report which methods were used to (statistically) compare the accuracy of tests so that the findings could be reproduced. Examples include McNemar’s test statistic for paired data, and tests for differences in the area under the receiver operating characteristic (ROC) curve. | Adapted from item 14 of STARD 2015 (1) |
| ***Participant flow and characteristics*** | |  |
| 1. Reporting a participant flow diagram, including all index tests | A flow diagram allows readers to identify shortcomings in study design. Ideally, the diagram should include all index tests in the comparison. | Adapted from item 19 of STARD 2015 (1) |
| 1. Reporting the baseline characteristics (at least age and gender) of participants for each index test group | Readers should be able to assess the similarity of participants allocated to each index test group with regards to factors that may affect test accuracy. | Adapted from item 15 of CONSORT 2010 (2) |
| **Results of the comparison** | | |
| ***Contingency table data*** | |  |
| 1. Reporting the two-by-two contingency table data for each index test | Such data can be used to reproduce the accuracy estimate for each index test. Data should be reported for each index test in the comparison. | Adapted from item 23 of STARD 2015 (1) |
| 1. If participants received multiple index tests, reporting the two-by-four contingency table data | Two-by-four tables (that cross-classify the results of one index test with another) allow readers to reproduce study results. Furthermore, such data allow for joint meta-analysis of studies that compare index tests on the same participants. | Item generated for this review |
| ***Comparative accuracy estimates*** | |  |
| 1. Reporting the results using comparative accuracy measures | While it may be sufficient to report the accuracy of each index test, comparative measures (such as absolute difference in sensitivity) may facilitate interpretation. | Item generated for this review |
| 1. Reporting measures of precision for comparative accuracy | Allows readers to appreciate the statistical uncertainty associated with the comparative accuracy estimate. | Item generated for this review |
| ***Limitations*** | |  |
| 1. Reporting any limitations regarding the comparison | While accuracy studies may describe limitations affecting the accuracy of individual tests, limitations in the test comparison may be overlooked. | Adapted from item 26 of STARD 2015 (1) |

**Table S3: List of included comparative accuracy studies.**

| # | Reference |
| --- | --- |
| 1 | Abdelhafez Y, Tawakol A, Osama A, Hamada E, El-Refaei S. Role of 18F-FDG PET/CT in the detection of ovarian cancer recurrence in the setting of normal tumor markers. Egypt J Radiol Nucl Med [Internet]. 2016;47(4):1787–94. Available from: http://www.sciencedirect.com/science/article/pii/S0378603X16301589 |
| 2 | Abdel-Rahman HM, El Fiki IM, Desoky EAE, Elsayed ER, Abd Samad KM. The role of diffusion-weighted magnetic resonance imaging in T staging and grading of urinary bladder cancer. Egypt J Radiol Nucl Med [Internet]. 2015;46(3):741–7. Available from: http://www.sciencedirect.com/science/article/pii/S0378603X15000637 |
| 3 | Agorastos T, Chatzistamatiou K, Katsamagkas T, Koliopoulos G, Daponte A, Constantinidis T, et al. Primary screening for cervical cancer based on high-risk human papillomavirus (HPV) detection and HPV 16 and HPV 18 genotyping, in comparison to cytology. PLoS One. 2015;10(3):e0119755. |
| 4 | Aida Y, Abe H, Tomita Y, Nagano T, Seki N, Sugita T, et al. Serum immunoreactive collagen IV detected by monoclonal antibodies as a marker of severe fibrosis in patients with non-alcoholic fatty liver disease. J Gastrointestin Liver Dis. 2015 Mar;24(1):61–8. |
| 5 | An YY, Kim SH, Kang BJ, Lee AW. Treatment Response Evaluation of Breast Cancer after Neoadjuvant Chemotherapy and Usefulness of the Imaging Parameters of MRI and PET/CT. J Korean Med Sci. 2015 Jun;30(6):808–15. |
| 6 | Azizi G, Keller JM, Mayo ML, Piper K, Puett D, Earp KM, et al. Thyroid nodules and shear wave elastography: A new tool in thyroid cancer detection. Ultrasound Med Biol. 2015;41(11):2855–65. |
| 7 | Bonnard P, Elsharkawy A, Zalata K, Delarocque-Astagneau E, Biard L, Le Fouler L, et al. Comparison of liver biopsy and noninvasive techniques for liver fibrosis assessment in patients infected with HCV-genotype 4 in Egypt. J Viral Hepat. 2015 Mar;22(3):245–53. |
| 8 | Boursier J, Vergniol J, Guillet A, Hiriart J-B, Lannes A, Le Bail B, et al. Diagnostic accuracy and prognostic significance of blood fibrosis tests and liver stiffness measurement by FibroScan in non-alcoholic fatty liver disease. J Hepatol. 2016 Sep;65(3):570–8. |
| 9 | Carrara S, Anderloni A, Jovani M, Di Tommaso L, Rahal D, Hassan C, et al. A prospective randomized study comparing 25-G and 22-G needles of a new platform for endoscopic ultrasound-guided fine needle aspiration of solid masses. Dig Liver Dis [Internet]. 2016;48(1):49–54. Available from: http://dx.doi.org/10.1016/j.dld.2015.09.017 |
| 10 | Chiang S-F, Kan C-Y, Hsiao Y-C, Tang R, Hsieh L-L, Chiang J-M, et al. Bone Marrow Stromal Antigen 2 Is a Novel Plasma Biomarker and Prognosticator for  Colorectal Carcinoma: A Secretome-Based Verification Study. Dis Markers. 2015;2015:874054. |
| 11 | Chimparlee N, Chuaypen N, Khlaiphuengsin A, Pinjaroen N, Payungporn S, Poovorawan Y, et al. Diagnostic and Prognostic Roles of Serum Osteopontin and Osteopontin Promoter Polymorphisms in Hepatitis B-related Hepatocellular Carcinoma. Asian Pac J Cancer Prev. 2015;16(16):7211–7. |
| 12 | Choi SH, Byun JH, Lim Y-S, Yu E, Lee SJ, Kim SY, et al. Diagnostic criteria for hepatocellular carcinoma 3 cm with hepatocyte-specific contrast-enhanced magnetic resonance imaging. J Hepatol. 2016 May;64(5):1099–107. |
| 13 | Chung SR, Lee SS, Kim N, Yu ES, Kim E, Kuhn B, et al. Intravoxel incoherent motion MRI for liver fibrosis assessment: a pilot study. Acta Radiol. 2015 Dec;56(12):1428–36. |
| 14 | Cui J, Ang B, Haufe W, Hernandez C, Verna EC, Sirlin CB, et al. Comparative diagnostic accuracy of magnetic resonance elastography vs. eight clinical prediction rules for non-invasive diagnosis of advanced fibrosis in biopsy-proven non-alcoholic fatty liver disease: A prospective study. Aliment Pharmacol Ther. 2015;41(12):1271–80. |
| 15 | Cui J, Heba E, Hernandez C, Haufe W, Hooker J, Andre MP, et al. Magnetic resonance elastography is superior to acoustic radiation force impulse for the Diagnosis of fibrosis in patients with biopsy-proven nonalcoholic fatty liver disease: A prospective study. Hepatology. 2016 Feb;63(2):453–61. |
| 16 | Da Costa AN, Plymoth A, Santos-Silva D, Ortiz-Cuaran S, Camey S, Guilloreau P, et al. Osteopontin and latent-TGF β binding-protein 2 as potential diagnostic markers for HBV-related hepatocellular carcinoma. Int J Cancer. 2015;136(1):172–81. |
| 17 | Dai L, Tsay J-CJ, Li J, Yie T-A, Munger JS, Pass H, et al. Autoantibodies against tumor-associated antigens in the early detection of lung cancer. Lung Cancer. 2016 Sep;99:172–9. |
| 18 | Deirmengian C, Kardos K, Kilmartin P, Cameron A, Schiller K, Booth REJ, et al. The alpha-defensin test for periprosthetic joint infection outperforms the leukocyte esterase test strip. Clin Orthop Relat Res. 2015 Jan;473(1):198–203. |
| 19 | Didier RA, Vajtai PL, Hopkins KL. Iterative reconstruction technique with reduced volume CT dose index: diagnostic accuracy in pediatric acute appendicitis. Pediatr Radiol [Internet]. 2014/07/05. 2015 Feb;45(2):181–7. Available from: https://www.ncbi.nlm.nih.gov/pubmed/24996812 |
| 20 | Ding D, Li H, Liu P, Chen L, Kang J, Zhang Y, et al. FibroScan, aspartate aminotransferase and alanine aminotransferase ratio (AAR), aspartate aminotransferase to platelet ratio index (APRI), fibrosis index based on the 4 factor (FIB-4), and their combinations in the assessment of liver fibrosis in patients. Int J Clin Exp Med. 2015;8(11):20876–82. |
| 21 | Dou S, Bai Y, Shandil A, Ding D, Shi D, Haacke EM, et al. Detecting prostate cancer and prostatic calcifications using advanced magnetic resonance imaging. Asian J Androl. 2017;19(4):439–43. |
| 22 | Drejer D, Béji S, Oezeke R, Nielsen AM, Høyer S, Bjerklund Johansen TE, et al. Comparison of White Light, Photodynamic Diagnosis, and Narrow-band Imaging in Detection of Carcinoma In Situ or Flat Dysplasia at Transurethral Resection of the Bladder: the DaBlaCa-8 Study. Urology [Internet]. 2017;102:138–42. Available from: http://dx.doi.org/10.1016/j.urology.2016.11.032 |
| 23 | Esiwe C, Baillon S, Rajkonwar A, Lindesay J, Lo N, Dennis M. Screening for depression in older adults on an acute medical ward: The validity of NICE guidance in using two questions. Age Ageing. 2015;44(5):771–5. |
| 24 | Eszlinger M, Piana S, Moll A, Bösenberg E, Bisagni A, Ciarrocchi A, et al. Molecular Testing of Thyroid Fine-Needle Aspirations Improves Presurgical Diagnosis and Supports the Histologic Identification of Minimally Invasive Follicular Thyroid Carcinomas. Thyroid. 2015;25(4):401–9. |
| 25 | Fang Z, Tang J, Bai Y, Lin H, You H, Jin H, et al. Plasma levels of microRNA-24, microRNA-320a, and microRNA-423-5p are potential biomarkers for colorectal carcinoma. J Exp Clin Cancer Res. 2015 Aug;34:86. |
| 26 | Feng Z-Y, Wang L, Min X-D, Wang S-G, Wang G-P, Cai J. Prostate Cancer Detection with Multiparametric Magnetic Resonance Imaging: Prostate Imaging Reporting and Data System Version 1 versus Version 2. Chin Med J (Engl) [Internet]. 2016 Oct 20;129(20):2451–9. Available from: https://www.ncbi.nlm.nih.gov/pubmed/27748338 |
| 27 | Fouad SA, Mohamed NAG, Fawzy MW, Moustafa DA. Plasma Osteopontin Level in Chronic Liver Disease and Hepatocellular Carcinoma. Hepat Mon. 2015 Sep;15(9):e30753. |
| 28 | Frangiamore SJ, Gajewski ND, Saleh A, Farias-Kovac M, Barsoum WK, Higuera CA. alpha-Defensin Accuracy to Diagnose Periprosthetic Joint Infection-Best Available Test? J Arthroplasty. 2016 Feb;31(2):456–60. |
| 29 | Frangiamore SJ, Siqueira MBP, Saleh A, Daly T, Higuera CA, Barsoum WK. Synovial Cytokines and the MSIS Criteria Are Not Useful for Determining Infection Resolution After Periprosthetic Joint Infection Explantation. Clin Orthop Relat Res. 2016 Jul;474(7):1630–9. |
| 30 | Galvin L, Oldan JD, Bahl M, Eastwood JD, Sosa JA, Hoang JK. Parathyroid 4D CT and Scintigraphy: What Factors Contribute to Missed Parathyroid Lesions? Otolaryngol Head Neck Surg. 2016 May;154(5):847–53. |
| 31 | Ginsburg M, Christoforidis GA, Zivin SP, Obara P, Wroblewski K, Angelos P, et al. Adenoma localization for recurrent or persistent primary hyperparathyroidism using dynamic four-dimensional CT and venous sampling. J Vasc Interv Radiol. 2015 Jan;26(1):79–86. |
| 32 | Giovanella L, Campenni A, Treglia G, Verburg FA, Trimboli P, Ceriani L, et al. Molecular imaging with (99m)Tc-MIBI and molecular testing for mutations in differentiating benign from malignant follicular neoplasm: a prospective comparison. Eur J Nucl Med Mol Imaging. 2016 Jun;43(6):1018–26. |
| 33 | Gu Y-L, Lan C, Pei H, Yang S-N, Liu Y-F, Xiao L-L. Applicative Value of Serum CA19-9, CEA, CA125 and CA242 in Diagnosis and Prognosis for Patients with Pancreatic Cancer Treated by Concurrent Chemoradiotherapy. Asian Pac J Cancer Prev. 2015;16(15):6569–73. |
| 34 | He F-C, Meng W-W, Qu Y-H, Zhou M-X, He J, Lv P, et al. Expression of circulating microRNA-20a and let-7a in esophageal squamous cell carcinoma. World J Gastroenterol [Internet]. 2015/04/21. 2015 Apr 21;21(15):4660–5. Available from: https://www.ncbi.nlm.nih.gov/pubmed/25914476 |
| 35 | Iftner T, Becker S, Neis K-J, Castanon A, Iftner A, Holz B, et al. Head-to-Head Comparison of the RNA-Based Aptima Human Papillomavirus (HPV) Assay  and the DNA-Based Hybrid Capture 2 HPV Test in a Routine Screening Population of Women Aged 30 to 60 Years in Germany. J Clin Microbiol. 2015 Aug;53(8):2509–16. |
| 36 | Ikematsu H, Matsuda T, Osera S, Imajoh M, Kadota T, Morimoto H, et al. Usefulness of narrow-band imaging with dual-focus magnification for differential  diagnosis of small colorectal polyps. Surg Endosc. 2015 Apr;29(4):844–50. |
| 37 | Jatuworapruk K, Lhakum P, Pattamapaspong N, Kasitanon N, Wangkaew S, Louthrenoo W. Performance of the Existing Classification Criteria for Gout in Thai Patients Presenting With Acute Arthritis. Medicine (Baltimore). 2016 Feb;95(5):e2730. |
| 38 | Jia J, Hou J, Ding H, Chen G, Xie Q, Wang Y, et al. Transient elastography compared to serum markers to predict liver fibrosis in a cohort of Chinese patients with chronic hepatitis B. J Gastroenterol Hepatol. 2015 Apr;30(4):756–62. |
| 39 | Jokela P, Vuorinen T, Waris M, Manninen R. Performance of the Alere i influenza A&B assay and mariPOC test for the rapid detection of influenza A and B viruses. J Clin Virol. 2015 Sep;70:72–6. |
| 40 | Kata SG, Aboumarzouk OM, Zreik A, Somani B, Ahmad S, Nabi G, et al. Photodynamic diagnostic ureterorenoscopy: A valuable tool in the detection of upper urinary tract tumour. Photodiagnosis Photodyn Ther [Internet]. 2016;13:255–60. Available from: http://dx.doi.org/10.1016/j.pdpdt.2015.08.002 |
| 41 | Khanna A, Khanna M, Gill KS. Comparative Evaluation of Tubex TF (Inhibition Magnetic Binding Immunoassay) for Typhoid Fever in Endemic Area. J Clin Diagn Res [Internet]. 2015/11/01. 2015 Nov;9(11):DC14-DC17. Available from: https://www.ncbi.nlm.nih.gov/pubmed/26676104 |
| 42 | Kumar N, Singh MK, Dayal R, Gupta S, Garg R. Diagnostic value of IL-6 in Neonatal sepsis. Ann Appl Bio-Sciences [Internet]. 2016;3(1):A67-71. Available from: http://www.pacificejournals.com/journal/index.php/aabs/article/view/aabs692 |
| 43 | Kwon YM, Antoci V, Leone WA, Tsai TY, Dimitriou D, Liow MHL. Utility of Serum Inflammatory and Synovial Fluid Counts in the Diagnosis of Infection in Taper Corrosion of Dual Taper Modular Stems. J Arthroplasty [Internet]. 2016;31(9):1997–2003. Available from: http://dx.doi.org/10.1016/j.arth.2016.02.020 |
| 44 | Lee MC, Gonzalez SJ, Lin H, Zhao X, Kiluk J V, Laronga C, et al. Prospective trial of breast MRI versus 2D and 3D ultrasound for evaluation of response to neoadjuvant chemotherapy. Ann Surg Oncol. 2015 Sep;22(9):2888–94. |
| 45 | Li Y-L, Zhang X-P, Li J, Cao K, Cui Y, Li X-T, et al. MRI in diagnosis of pathological complete response in breast cancer patients after neoadjuvant chemotherapy. Eur J Radiol [Internet]. 2015 Feb 1;84(2):242–9. Available from: https://doi.org/10.1016/j.ejrad.2014.11.029 |
| 46 | Lin Q, Lim HSR, Lin HL, Tan HT, Lim TK, Cheong WK, et al. Analysis of colorectal cancer glyco-secretome identifies laminin beta-1 (LAMB1) as a potential serological biomarker for colorectal cancer. Proteomics. 2015 Nov;15(22):3905–20. |
| 47 | Lin WC, Muglia VF, Silva GEB, Filho SC, Reis RB, Westphalen AC. Multiparametric MRI of the prostate: Diagnostic performance and interreader agreement of two scoring systems. Br J Radiol. 2016;89(1062). |
| 48 | Löffler C, Sattler H, Peters L, Löffler U, Uppenkamp M, Bergner R. Distinguishing gouty arthritis from Calcium Pyrophosphate disease and other arthritides. J Rheumatol. 2015;42(3):513–20. |
| 49 | Lu S, Gao Q, Yu J, Li Y, Cao P, Shi H, et al. Utility of dynamic contrast-enhanced magnetic resonance imaging for differentiating glioblastoma, primary central nervous system lymphoma and brain metastatic tumor. Eur J Radiol. 2016 Oct;85(10):1722–7. |
| 50 | Ma C, Liu Y, Liu X, Yin F, Lu Q. Comparison of Different Screening Methods for Hypertension in Han Adolescents. Clin Pediatr (Phila). 2016 Apr;55(4):363–7. |
| 51 | Magee T. Comparison of 3.0-T MR vs 3.0-T MR arthrography of the hip for detection of acetabular labral tears and chondral defects in the same patient population. Br J Radiol. 2015;88(1053):1–7. |
| 52 | Manganaro L, D’Ambrosio I, Gigli S, Di Pastena F, Giraldi G, Tardioli S, et al. Breast MRI in patients with unilateral bloody and serous-bloody nipple discharge: a comparison with galactography. Biomed Res Int. 2015;2015:806368. |
| 53 | Mansoor S, Yerian L, Kohli R, Xanthakos S, Angulo P, Ling S, et al. The evaluation of hepatic fibrosis scores in children with nonalcoholic fatty liver disease. Dig Dis Sci. 2015 May;60(5):1440–7. |
| 54 | Meissnitzer T, Seymer A, Keinrath P, Holzmannhofer J, Pirich C, Hergan K, et al. Added value of semi-quantitative breast-specific gamma imaging in the work-up of  suspicious breast lesions compared to mammography, ultrasound and 3-T MRI. Br J Radiol. 2015 Jul;88(1051):20150147. |
| 55 | Mirghani H, Casiraghi O, Amen F, He M, Ma X-J, Saulnier P, et al. Diagnosis of HPV-driven head and neck cancer with a single test in routine clinical practice. Mod Pathol  an Off J United States Can Acad  Pathol Inc. 2015 Dec;28(12):1518–27. |
| 56 | Mourato FA, Lima Filho JL, Mattos SDS. Comparison of different screening methods for blood pressure disorders in children and adolescents. J Pediatr (Rio J) [Internet]. 2015;91(3):278–83. Available from: http://dx.doi.org/10.1016/j.jped.2014.08.008 |
| 57 | Nasr P, Hilliges A, Thorelius L, Kechagias S, Ekstedt M. Contrast-enhanced ultrasonography could be a non-invasive method for differentiating none or mild from severe fibrosis in patients with biopsy proven non-alcoholic fatty liver disease. Scand J Gastroenterol. 2016 Sep;51(9):1126–32. |
| 58 | Noh JY, Choi WS, Lee J, Kim HL, Song JY, Cheong HJ, et al. Clinical performance of the SofiaTM Influenza A+B FIA in adult patients with influenza-like illness. Diagn Microbiol Infect Dis. 2015;83(2):130–2. |
| 59 | Norton ME, Jacobsson B, Swamy GK, Laurent LC, Ranzini AC, Brar H, et al. Cell-free DNA analysis for noninvasive examination of trisomy. N Engl J Med. 2015 Apr;372(17):1589–97. |
| 60 | Önür ST, Sökücü SN, Dalar L, Seyhan EC, Akbaş A, Altin S. Are soluble IL-2 receptor and IL-12p40 levels useful markers for diagnosis of tuberculous pleurisy? Infect Dis (Auckl). 2015;47(3):150–5. |
| 61 | Park JJ, Park BK. Role of PI-RADSv2 with multiparametric MRI in determining who needs active surveillance or definitive treatment according to PRIAS. J Magn Reson Imaging. 2017;45(6):1753–9. |
| 62 | Paspulati RM, Partovi S, Herrmann KA, Krishnamurthi S, Delaney CP, Nguyen NC. Comparison of hybrid FDG PET/MRI compared with PET/CT in colorectal cancer staging and restaging: a pilot study. Abdom Imaging. 2015 Aug;40(6):1415–25. |
| 63 | Petrillo M, Fusco R, Catalano O, Sansone M, Avallone A, Delrio P, et al. MRI for Assessing Response to Neoadjuvant Therapy in Locally Advanced Rectal Cancer Using DCE-MR and DW-MR Data Sets: A Preliminary Report. Biomed Res Int. 2015;2015:514740. |
| 64 | Powrózek T, Krawczyk P, Kowalski DM, Kuźnar-Kamińska B, Winiarczyk K, Olszyna-Serementa M, et al. Application of plasma circulating microRNA-448, 506, 4316, and 4478 analysis for non-invasive diagnosis of lung cancer. Tumor Biol. 2016;37(2):2049–55. |
| 65 | Prasad KJ, Oberoi JK, Goel N, Wattal C. Comparative evaluation of two rapid Salmonella-IgM tests and blood culture in the diagnosis of enteric fever. Indian J Med Microbiol. 2015;33(2):237–42. |
| 66 | Queiroz MA, Kubik-Huch RA, Hauser N, Freiwald-Chilla B, von Schulthess G, Froehlich JM, et al. PET/MRI and PET/CT in advanced gynaecological tumours: initial experience and comparison. Eur Radiol. 2015 Aug;25(8):2222–30. |
| 67 | Radford K, Mack HA, Draper B, Chalkley S, Delbaere K, Daylight G, et al. Comparison of Three Cognitive Screening Tools in Older Urban and Regional Aboriginal Australians. Dement Geriatr Cogn Disord. 2015;40(1–2):22–32. |
| 68 | Reechaipichitkul W, Pimrin W, Bourpoern J, Prompinij S, Faksri K. Evaluation of the QuantiFERON®-TB gold in-tube assay and tuberculin skin test for the diagnosis of Mycobacterium tuberculosis infection in northeastern Thailand. Asian Pacific J Allergy Immunol. 2015;33(3):236–44. |
| 69 | Reisæter LA, Fütterer JJ, Halvorsen OJ, Nygård Y, Biermann M, Andersen E, et al. 1.5-T multiparametric MRI using PI-RADS: A region by region analysis to localize the index-tumor of prostate cancer in patients undergoing prostatectomy. Acta radiol. 2015;56(4):500–11. |
| 70 | Ryu SW, Suh IB, Ryu S-M, Shin KS, Kim H-S, Kim J, et al. Comparison of three rapid influenza diagnostic tests with digital readout systems and one conventional rapid influenza diagnostic test. J Clin Lab Anal. 2018 Feb;32(2). |
| 71 | Saha A, Mukhopadhyay M, Das C, Sarkar K, Saha AK, Sarkar DK. FNAC Versus Core Needle Biopsy: A Comparative Study in Evaluation of Palpable Breast Lump. J Clin Diagn Res [Internet]. 2016/02/01. 2016 Feb;10(2):EC05-EC8. Available from: https://www.ncbi.nlm.nih.gov/pubmed/27042469 |
| 72 | Sawicki LM, Grueneisen J, Schaarschmidt BM, Buchbender C, Nagarajah J, Umutlu L, et al. Evaluation of (1)(8)F-FDG PET/MRI, (1)(8)F-FDG PET/CT, MRI, and CT in whole-body  staging of recurrent breast cancer. Eur J Radiol. 2016 Feb;85(2):459–65. |
| 73 | Schaefgen B, Mati M, Sinn HP, Golatta M, Stieber A, Rauch G, et al. Can Routine Imaging After Neoadjuvant Chemotherapy in Breast Cancer Predict Pathologic Complete Response? Ann Surg Oncol. 2016 Mar;23(3):789–95. |
| 74 | Schwartz RH, Selvarangan R, Zissman EN. BD Veritor System Respiratory Syncytial Virus Rapid Antigen Detection Test. Pediatr Emerg Care. 2015;31(12):830–4. |
| 75 | Seeburger JL, Holder DJ, Combrinck M, Joachim C, Laterza O, Tanen M, et al. Cerebrospinal fluid biomarkers distinguish postmortem-confirmed Alzheimer’s disease from other dementias and healthy controls in the OPTIMA cohort. J Alzheimers Dis. 2015;44(2):525–39. |
| 76 | Seo YS, Kim MY, Kim SU, Hyun BS, Jang JY, Lee JW, et al. Accuracy of transient elastography in assessing liver fibrosis in chronic viral hepatitis: A multicentre, retrospective study. Liver Int. 2015 Oct;35(10):2246–55. |
| 77 | Serna A, Contador I, Bermejo-Pareja F, Mitchell AJ, Fernandez-Calvo B, Ramos F, et al. Accuracy of a Brief Neuropsychological Battery for the Diagnosis of Dementia and  Mild Cognitive Impairment: An Analysis of the NEDICES Cohort. J Alzheimers Dis. 2015;48(1):163–73. |
| 78 | Shagos GS, Shanmugasundaram P, Varma AK, Padma S, Sarma M. 18-F flourodeoxy glucose positron emission tomography-computed tomography imaging: A viable alternative to three phase bone scan in evaluating diabetic foot complications? Indian J Nucl Med [Internet]. 2015;30(2):97–103. Available from: https://www.ncbi.nlm.nih.gov/pubmed/25829725 |
| 79 | Shemmassian SK, Lee SS. Predictive Utility of Four Methods of Incorporating Parent and Teacher Symptom Ratings of ADHD for Longitudinal Outcomes. J Clin Child Adolesc Psychol [Internet]. 2016;45(2):176–87. Available from: http://dx.doi.org/10.1080/15374416.2014.971457 |
| 80 | Sheridan K, Kreulen C, Kim S, Mak W, Lewis K, Marder R. Accuracy of magnetic resonance imaging to diagnose superior labrum anterior-posterior tears. Knee Surg Sports Traumatol Arthrosc. 2015 Sep;23(9):2645–50. |
| 81 | Shi Y, Xia F, Li Q-J, Li J-H, Yu B, Li Y, et al. Magnetic Resonance Elastography for the Evaluation of Liver Fibrosis in Chronic Hepatitis B and C by Using Both Gradient-Recalled Echo and Spin-Echo Echo Planar Imaging: A Prospective Study. Am J Gastroenterol. 2016 Jun;111(6):823–33. |
| 82 | Sonawane V. Study of Interleukin-6 Levels in Early Diagnosis of Neonatal Sepsis. Int J Res Med Sci. 2015;3(1):1. |
| 83 | Song PH, Cho S, Ko YH. Decision Based on Narrow Band Imaging Cystoscopy without a Referential Normal Standard Rather Increases Unnecessary Biopsy in Detection of Recurrent Bladder Urothelial Carcinoma Early after Intravesical Instillation. Cancer Res Treat [Internet]. 2015/03/02. 2016 Jan;48(1):273–80. Available from: https://www.ncbi.nlm.nih.gov/pubmed/25761489 |
| 84 | Sun H, Shi J-X, Zhang H-F, Xing M-T, Li P, Dai L-P, et al. Serum autoantibodies against a panel of 15 tumor-associated antigens in the detection of ovarian cancer. Tumour Biol. 2017 Jun;39(6):1010428317699132. |
| 85 | Taguchi A, Rho J-H, Yan Q, Zhang Y, Zhao Y, Xu H, et al. MAPRE1 as a plasma biomarker for early-stage colorectal cancer and adenomas. Cancer Prev Res (Phila). 2015 Nov;8(11):1112–9. |
| 86 | Tan N, Lin W-C, Khoshnoodi P, Asvadi NH, Yoshida J, Margolis DJA, et al. In-Bore 3-T MR-guided Transrectal Targeted Prostate Biopsy: Prostate Imaging Reporting and Data System Version 2-based Diagnostic Performance for Detection of Prostate Cancer. Radiology. 2017 Apr;283(1):130–9. |
| 87 | Taylor WJ, Fransen J, Dalbeth N, Neogi T, Schumacher HR, Brown M, et al. Performance of classification criteria for gout in early and established disease. Ann Rheum Dis [Internet]. 2016 Jan;75(1):178–82. Available from: http://ard.bmj.com/lookup/doi/10.1136/annrheumdis-2014-206364 |
| 88 | Tikku G, Umap P. Comparative study of core needle biopsy and fine needle aspiration cytology in palpable breast lumps: Scenario in developing nations. Turk Patoloji Derg. 2016;32(1):1–7. |
| 89 | Tuerxun G, Yukesaier A, Lu L, Aierken K, Mijiti P, Jiang Y, et al. Evaluation of careHPV, Cervista Human Papillomavirus, and Hybrid Capture 2 Methods in Diagnosing Cervical Intraepithelial Neoplasia Grade 2+ in Xinjiang Uyghur Women. Oncologist. 2016;21(7):825–31. |
| 90 | Tuttle R, Weick A, Schwarz WS, Chen X, Obermeier P, Seeber L, et al. Evaluation of novel second-generation RSV and influenza rapid tests at the point of care. Diagn Microbiol Infect Dis [Internet]. 2015;81(3):171–6. Available from: http://dx.doi.org/10.1016/j.diagmicrobio.2014.11.013 |
| 91 | Van Der Hoeven EJRJ, Dankbaar JW, Algra A, Vos JA, Niesten JM, Van Seeters T, et al. Additional Diagnostic Value of Computed Tomography Perfusion for Detection of Acute Ischemic Stroke in the Posterior Circulation. Stroke. 2015;46(4):1113–5. |
| 92 | Wang H-J, Pui MH, Guan J, Li S-R, Lin J-H, Pan B, et al. Comparison of Early Submucosal Enhancement and Tumor Stalk in Staging Bladder Urothelial Carcinoma. AJR Am J Roentgenol. 2016 Oct;207(4):797–803. |
| 93 | Wang RJ, Zheng YH, Wang P, Zhang JZ. Serum miR-125a-5p, miR-145 and miR-146a as diagnostic biomarkers in non-small cell lung cancer. Int J Clin Exp Pathol. 2015;8(1):765–71. |
| 94 | Wang X, Zhi X, Zhang Y, An G, Feng G. Role of plasma MicroRNAs in the early diagnosis of non-small-cell lung cancers: a case-control study. J Thorac Dis. 2016 Jul;8(7):1645–52. |
| 95 | Wu C-H, Ho M-C, Jeng Y-M, Liang P-C, Hu R-H, Lai H-S, et al. Assessing hepatic fibrosis: comparing the intravoxel incoherent motion in MRI with acoustic radiation force impulse imaging in US. Eur Radiol. 2015 Dec;25(12):3552–9. |
| 96 | Yamashita K, Hiwatashi A, Togao O, Kikuchi K, Kitamura Y, Mizoguchi M, et al. Diagnostic utility of intravoxel incoherent motion mr imaging in differentiating  primary central nervous system lymphoma from glioblastoma multiforme. J Magn Reson Imaging. 2016 Nov;44(5):1256–61. |
| 97 | Zaporozhchenko IA, Morozkin ES, Skvortsova TE, Ponomaryova AA, Rykova EY, Cherdyntseva N V, et al. Plasma miR-19b and miR-183 as Potential Biomarkers of Lung Cancer. PLoS One [Internet]. 2016 Oct 21;11(10):e0165261–e0165261. Available from: https://www.ncbi.nlm.nih.gov/pubmed/27768748 |
| 98 | Zhang Y, Liu Y-J, Liu T, Zhang H, Yang S-J. Plasma microRNA-21 is a potential diagnostic biomarker of acute myocardial infarction. Eur Rev Med Pharmacol Sci. 2016;20(2):323–9. |
| 99 | Zhou C, Chen Z, Dong J, Li J, Shi X, Sun N, et al. Combination of serum miRNAs with Cyfra21-1 for the diagnosis of non-small cell lung cancer. Cancer Lett. 2015 Oct;367(2):138–46. |
| 100 | Zhu WY, Zhou KY, Zha Y, Chen DD, He JY, Ma HJ, et al. Diagnostic Value of Serum miR-182, miR-183, miR-210, and miR-126 levels in patients with early-stage non-small cell lung cancer. PLoS One. 2016;11(4):1–16. |

**References**

1. Bossuyt PM, Reitsma JB, Bruns DE, Gatsonis CA, Paul P, Irwig L, et al. STARD 2015: an updated list of essential items for reporting diagnostic accuracy studies. BMJ Br Med J. 2015;351.
2. Schulz KF, Altman DG, Moher D. CONSORT 2010 Statement: Updated guidelines for reporting parallel group randomised trials. BMJ. 2010;340(7748):698–702.
3. Yang B. QUADAS-2C Delphi Process [Internet]. OSF; 2019. Available from: osf.io/tmze9
